# Diagnosing Urinary Tract Infection in Young Febrile Children in the Emergency Department

**DOI:** 10.1001/jamanetworkopen.2026.1741

**Published:** 2026-03-13

**Authors:** Ceilidh Kinlin, Jocelyn Gravel, Nick Barrowman, Vid Bijelik, Ramona Cook, Natasha Wills-Ibarra, Roger Zemek, Nader Shaikh, Maala Bhatt

**Affiliations:** 1 Department of Pediatrics, Children’s Hospital of Eastern Ontario, University of Ottawa, Ottawa, Ontario, Canada; 2 Department of Pediatric Emergency Medicine, Centre Hospitalier Universitaire Sainte-Justine, Université de Montréal, Montréal, Quebec, Canada; 3 Children’s Hospital of Eastern Ontario Research Institute, Ottawa, Ontario, Canada; 4 Department of Pediatrics and Emergency Medicine, Children’s Hospital of Eastern Ontario Research Institute, University of Ottawa, Ottawa, Ontario, Canada; 5 University of Pittsburgh School of Medicine, Pittsburgh, Pennsylvania

## Abstract

**Importance:**

Diagnosing urinary tract infection (UTI) in preverbal, pre–toilet trained children is challenging and may lead to unnecessary testing and treatment. UTICalc estimates UTI risk using clinical and laboratory data but has not been prospectively validated.

**Objectives:**

To prospectively validate UTICalc version 3.0 for predicting UTI and to evaluate its utility in guiding clinical decisions.

**Design, Setting, and Participants:**

This prospective diagnostic study was conducted at 2 tertiary care pediatric emergency departments (EDs) in Canada from November 2022 to January 2025. Children aged 2 to 24 months presenting with measured fever (≥38.0 °C) were enrolled. Exclusions included congenital urinary tract abnormalities, immunosuppression, current antimicrobials, or prior enrollment. Participants were followed up to identify UTIs; urine culture results were reviewed within 48 hours, and phone or email follow-up was completed within 2 weeks for those without testing at the index visit.

**Exposure:**

UTICalc-predicted probability of UTI.

**Main Outcomes and Measures:**

The primary outcome was UTI, defined as positive urinalysis and significant uropathogen culture growth. The primary analysis examined discriminative performance using area under receiver operating characteristic curve (AUROC). Secondary analyses included calibration (calibration plots, slope, Brier score) and clinical utility (net benefit analysis, comparison with clinician practice).

**Results:**

Among 2561 included participants (1212 [47%] female; 1326 [64%] younger than 12 months), 111 children (4%) were classified as having a UTI. In the full sample, 2256 (88.1%) had urine testing and/or provided follow-up data. The clinical model AUROC was 84.1% (95% CI, 80.4%-87.9%). At a 2% UTI risk threshold, sensitivity was 96.4%, and specificity was 34.1%; at a 5% risk threshold, sensitivity was 82.0%, and specificity was 73.8%. The clinical and dipstick model AUROC was 95.3% (95% CI, 93.3%-97.4%), with 94.0% sensitivity and 86.9% specificity at 5% risk. The sensitivity and specificity for clinicians were 98.2% and 57.3%, respectively. Calibration slopes were 0.11 (95% CI, 0.09-0.13) and 0.06 (95% CI, 0.05-0.07) and Brier scores were 0.04 (95% CI, 0.03-0.05) and 0.05 (95% CI, 0.04-0.06) for the clinical and clinical and dipstick models, respectively. Decision curve analysis showed both models were associated with positive net benefit across a range of risk thresholds.

**Conclusions and Relevance:**

In this cohort study of 2561 febrile children aged 2 to 24 months presenting to the ED, UTICalc showed strong diagnostic performance, especially with dipstick results. While it did not outperform experienced clinicians, it offered a useful, evidence-based adjunct for guiding urine testing decisions, supporting efficient care in the pediatric ED.

## Key Points

**Question:**

What is the diagnostic accuracy of UTICalc version 3.0 in predicting urinary tract infections (UTIs) for febrile children aged 2 to 24 months in the emergency department?

**Findings:**

In this diagnostic study with 2561 children, UTICalc showed strong discrimination, with an area under the receiver operating characteristic curve (AUROC) of 84.1% for the clinical model and an AUROC of 95.3% for the clinical and dipstick model.

**Meaning:**

In this study, UTICalc had high accuracy in identifying UTIs in young febrile children, which may help guide testing and treatment decisions in the emergency department.

## Teaser

This diagnostic study validates UTICalc version 3.0, a race-neutral prediction model for urinary tract infections in children, and evaluates its clinical utility among children aged 2 to 24 months presenting to the emergency department with fever.

## Introduction

Febrile illness accounts for 14% of children emergency department (ED) visits, with urinary tract infection (UTIs) responsible for as many as 7% of these cases.^1,2,3^ Diagnosing UTIs in preverbal and pre–toilet trained children is particularly challenging due to nonspecific symptoms and difficulty obtaining clean urine samples.^4^ In this population, urethral catheterization can cause patient discomfort and carries a risk of iatrogenic infection,^5,6^ and clean-catch methods are prone to contimination.^7^ Accurate diagnosis is critical; missed UTIs risk potential progression to urosepsis and may have long-term consequences such as kidney scarring,^8,9,10,11,12^ while overuse of antibiotics contributes to antimicrobial resistance.^13^

Over the years, researchers have studied this population to identify risk factors for UTIs, aiming to guide clinicians in risk stratification and inform decisions about testing and treatment.^11^ UTICalc is a clinical prediction tool designed to improve the diagnosis of UTIs in young children by estimating risk based on clinical and laboratory data.^14^ Initially developed using a nested case-control study, UTICalc version 1.0 demonstrated strong diagnostic performance (area under the receiver operating characteristic curve [AUROC], 0.81; 95% CI, 0.72-0.89), which improved further with the inclusion of dipstick results (AUROC, 0.99; 95% CI, 0.98-0.99).^14^ The tool is available via a web-based interface,^15^ where users input patient characteristics to calculate UTI risk.

To address concerns about racial bias in clinical decision-making, UTICalc underwent iterative updates that replaced race as a variable with duration of fever and history of UTI, while preserving its diagnostic performance.^16,17,18^ In 2023, version 3.0 introduced updated risk thresholds to guide testing decisions: testing is recommended for children with a calculated risk of greater than 5%, not recommended for a risk of less than 2%, and shared-decision making is encouraged for risks between 2% and 5%.^14,16^ These changes reflect the tool’s high sensitivity within the 2% to 5% risk threshold range and reinforce commitment to equitable, evidence-based care.

Despite widespread use and thoughtful evolution, UTICalc has only been retrospectively validated.^16,19^ Retrospective validation, while useful for initial assessment, is inherently limited by selection bias and missing data, which can lead to overestimation of a tool’s performance.^20^ Prospective validation is essential to evaluate the accuracy of UTICalc, its generalizability across diverse clinical settings, and its potential impact on clinical outcomes.^21^ This study aimed to provide a prospective external validation of UTICalc version 3.0 in children aged 2 to 24 months presenting to the pediatric ED with fever. The primary objective was to assess the model’s discriminative ability. Secondary objectives included evaluating its calibration and clinical utility.

## Methods

### Study Design and Settings

We conducted a multicenter prospective diagnostic study between November 2022 and January 2025 in 2 Canadian tertiary care pediatric EDs, both members of the Pediatric Emergency Research Canada (PERC) network^22^: Children’s Hospital of Eastern Ontario (CHEO) (Ottawa, Ontario) and Centre Hospitalier Universitaire Ste Justine (Montreal, Quebec). These are high-volume EDs, each with an annual census exceeding 70 000 visits. The study received approval from the research ethics board at both institutions. The legal guardian of all participants provided written informed consent prior to study enrollment. This study protocol was not registered in a public research repository. This study was reported in accordance Strengthening the Reporting of Observational Studies in Epidemiology (STROBE) and Transparent Reporting of a Multivariable Prediction Model for Individual Prognosis or Diagnosis (TRIPOD)^23^ reporting guidelines.

### Study Population

Children aged 2 to 24 months presenting with fever were eligible for enrollment. Fever was defined as a temperature of 38.0 °C higher, objectively measured in the ED or at home within the previous 24 hours by any method. Exclusion criteria included known congenital kidney or urinary tract abnormalities (including neurogenic bladder), oral or intravenous antibiotic use for any reason at the time of eligibility screening, immunosuppression, insurmountable language barrier, or prior enrollment in the study.

### Sample Size

Sample size calculations were based on calibration and precision criteria for external validation of a prediction model with a binary outcome.^24^ Assuming a C-statistic (AUROC) of 0.81, as reported in the original UTICalc derivation, with a standard error of 0.025, and standard errors of 0.10 for both calibration-in-the-large and calibration slope, we estimated the need for 118 UTI events.^14,24^ Given a UTI prevalence of 4.6% at the lead site, this translated to a total required sample size of 2561 participants.

### Study Protocol

Trained research staff were in the ED for up to 12 hours per day (9 am to 9 pm, 7 days a week) to screen and enroll eligible children. Research assistants prospectively screened patients for eligibility. For eligible patients, caregivers provided written informed consent for participation and contact information for follow-up. Decisions regarding urinalysis, urine culture, empiric antibiotic treatment, and ED disposition were made at the clinical team’s discretion according to usual care and were not influenced by study participation. Treating clinicians and caregivers were unaware of the final UTI status, which was determined using predefined urinalysis and urine culture criteria. Diagnostic and treatment decisions were made at the discretion of the treating team, including whether to use UTICalc. Customary practice at one ED was not to use UTICalc and varied at the second ED. Case predictions were not explicitly shared with the team.

### Data Collection and Outcome Measures

During the index visit, consenting caregivers completed a standardized electronic data collection form, capturing demographic and clinical characteristics for their child, including age, sex, circumcision status, fever duration, and maximum recorded temperature as well as prior history of UTI. The treating team completed a standardized paper data collection form with questions related to fever source. Within 1 week of the ED visit, medical record reviews were conducted to collect laboratory predictor variables and culture results. These were performed by 2 study personnel (N.W.-I. and R.C.), with approximately 5% independently reviewed by each site investigator (C.K. and J.G.) to ensure data quality.

If no urine sample was collected in the ED, email or phone follow-up was completed with caregivers to identify any UTI diagnoses made within 48 hours of the index visit. Questionnaires were sent 3 days after the visit, with 2 reminder messages at 3-day intervals. If there was no response, up to 3 follow-up phone calls were made, each spaced at least 24 hours apart. All study data were securely captured in REDCap, a secure online research program, and housed at CHEO.

Participants were classified as UTI positive if they met both of the following criteria: (1) urinalysis showing either any leukocyte esterase greater than trace on dipstick or greater than 5 white blood cells per high-power field on microscopy and (2) urine culture demonstrating growth of a single uropathogen at 10 × 10^7^ colony-forming units (CFU)/L or greater from a clean-void or clean-catch sample or 5 × 10^7^ CFU/L or greater from a catheter sample. Uropathogens included *Escherichia coli*, *Klebsiella* spp, *Proteus* spp, *Enterobacter* spp, *Enterococcus* spp, *Citrobacter* spp, and *Serratia marcescens*, consistent with published definitions.^4,25^ During medical record review, any case in which the treating clinician diagnosed a UTI despite not meeting the a priori criteria was flagged and reviewed by study personnel to reach consensus. Additionally, cases were considered UTI positive if a UTI was reported in the follow-up questionnaire. Participants were considered UTI negative if they met any of the following criteria: (1) no missed UTIs reported on the follow-up questionnaire, (2) urinalysis was negative and no urine culture was performed, or (3) a urine culture was performed but it did not meet the study definition for UTI.

UTICalc version 3.0 clinical model includes 5 categorical clinical risk factors (age <12 months, sex and circumcision status, temperature >39 °C, no other fever source, and duration of fever >48 hours). The clinical and dipstick model includes variables from the clinical model plus leukocyte esterase and nitrite values.

### Statistical Analysis

Participant characteristics were compared using descriptive statistics (frequency and percentage for categorical variables). Predicted probabilities of UTI were calculated a posteriori by the research team using the UTICalc version 3.0 algorithm, which was provided by the developing team and is based on previously published derivation and validation studies.^14,16^ Model parameters were applied to the clinical and laboratory data collected in our cohort without any recalibration.

Model performance was assessed by constructing a receiver operating characteristic (ROC) curve, illustrating model discrimination (sensitivity and specificity) across the full range of possible thresholds. The AUROC was then computed. Sensitivity, specificity, and positive and negative predictive values were reported using operational UTICalc version 3.0 risk thresholds (2% and 5% for the clinical model and 5% for the clinical and dipstick model).^14,16^ Calibration was evaluated using calibration plots comparing predicted probabilities with observed outcomes and quantified using the calibration slope, which reflects agreement between predicted and observed risks; the observed-to-expected ratio, which compares the number of observed events with the number of expected events by the model and for which a ratio of 1 would indicate perfect agreement; and the Brier score, which measures the mean squared difference between predicted probabilities and observed outcomes. Clinical utility was assessed using decision curve analysis to calculate the net benefit of the models across a range of threshold probabilities (defined as the minimum probability of UTI at which testing or treatment would be warranted) compared with default treat-all or treat-none strategies; a model with higher net benefit at a given threshold was considered clinically useful.^26^ We also compared UTICalc recommendation for urine testing to clinician practice.

Missing predictor variable data were handled by excluding incomplete cases. The potential impact of missing data was assessed by comparing observed characteristics between participants with and without missing information. All statistical analyses were performed using R version 4.4.1 (R Project for Statistical Computing), with a 2-sided significance level of α = .05.

## Results

### Participant Flow and Characteristics

**Figure 1.**
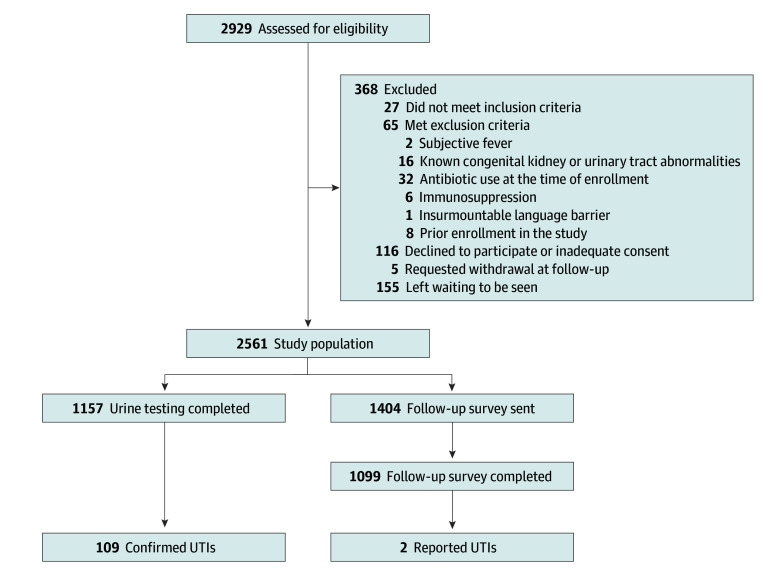
Participant Flow Diagram UTI indicates urinary tract infection.

Among 2561 included participants (1212 [47%] female; 1326 [64%] <12 months) (Figure 1 and Table 1), 111 children (4%) were classified as having UTI. Of these, 105 met the UTI-positive case definition, 2 were reported on follow-up questionnaire, and 4 were adjudicated as UTI-positive cases (Table 2). All adjudicated UTI-positive cases were infants with positive dipstick results and had significant growth of 2 uropathogens. Clinical and laboratory characteristics are summarized in Table 1, overall and by UTI status. Among children with UTI, 71 (64%) were younger than 12 months compared with 40 (36%) aged 12 to younger than 24 months. Female children represented 47% of the overall cohort (1204 of 2561) and 62% of UTI cases (69 of 111). Uncircumcised males represented 34% of the sample (870 children) and 35% of UTI cases (39 children), whereas circumcised males accounted for 19% of participants (487 children) and 3% of UTI cases (3 children).

**Table 1.** Participant Characteristics and Predictor Variables by UTI Outcome

Predictor variable	Participants. No. (%)		
	Overall (N = 2561)	UTI status	
		Positive (n = 111)	Negative (n = 2450)
Clinical characteristics			
Age, mo			
2 to <12	1326 (52)	71 (64)	1580 (64)
12 to <24	1235 (48)	40 (36)	870 (36)
Maximum temperature, °C			
<39	855 (33)	32 (29)	823 (34)
≥39	1706 (67)	79 (71)	1627 (66)
History of UTI			
Yes	133 (5)	9 (8)	124 (5)
No	2428 (95)	102 (92)	2326 (95)
Sex and circumcision status			
Female	1212 (47)	69 (62)	1143 (47)
Uncircumcised male	866 (34)	39 (35)	827 (34)
Circumcised male	479 (19)	3 (3)	476 (19)
Missing	4 (<1)	0	4 (<1)
Duration of fever, h			
<48	1129 (44)	45 (41)	1084 (44)
≥48	1432 (56)	66 (59)	1366 (56)
Other fever source			
Yes	2050 (81)	27 (24)	2023 (83)
Upper respiratory tract infection	1025 (50)	12 (44)	1013 (50)
Lower respiratory tract infection	246 (12)	3 (11)	243 (12)
Acute otitis media	452 (22)	4 (15)	448 (22)
Gastroenteritis	108 (5)	7 (26)	101 (5)
Other^a^	219 (11)	1 (4)	218 (11)
None	511 (19)	84 (76)	427 (17)
Dipstick results^b^			
No.	895	100	795
Nitrite			
Positive	41 (5)	33 (33)	8 (1)
Negative	854 (95)	77 (77)	787 (99)
Leukocyte esterase			
None	637 (71)	3 (3)	634 (80)
Trace	80 (9)	8 (8)	72 (9)
1+	81 (9)	24 (24)	57 (7)
2+	50 (6)	33 (33)	17 (2)
3+	47 (5)	32 (32)	15 (2)

Abbreviation: UTI, urinary tract infection.

^a^ Other sources of fever inclusive of specific infections, such as hand, foot and mouth disease, roseola, and adenitis, or multiple sources of fever identified by clinician (for example, upper respiratory tract infection and acute otitis media).

^b^ Only cases with predicted UTI risk greater than 2% who had urine dipstick results available were included in the model.

**Table 2.** Clinically Adjudicated Urinary Tract Infection–Positive Cases Not Meeting A Priori Definition

Clinical characteristics	Result	
	Dipstick	Urine culture
Uncircumcised male aged 4 mo with 18 h of fever, a maximum recorded temperature of 40.1 °C without a clear source of fever on history or examination	Nitrite negative 3+ Leukocyte esterase	≥10 × 10^7^ CFU/L *Klebsiella* pneumonia ≥10 × 10^7^ CFU/L extended spectrum beta lactamase *Escherichia coli*
Uncircumcised male aged 2 mo with 120 h of fever, a maximum recorded temperature of 39.8 °C without a clear source of fever on history or examination	Nitrite negative Trace leukocyte esterase	≥10 × 10^7^ CFU/L *Enterococcus faecaelis* ≥10 × 10^7^ CFU/L *Citrobacter freundii* complex
Female aged 6 mo with 72 h of fever, a maximum recorded temperature of 39.4 °C without a clear source of fever on history or examination	Nitrite negative 2+ Leukocyte esterase	≥10 × 10^7^ CFU/L *Klebsiella* pneumonia ≥10 × 10^7^ CFU/L *Escherichia coli*
Female aged 4 mo with 120 h of fever, a maximum recorded temperature of 40.2 °C without a clear source of fever on history or examination	Nitrite negative 3+ Leukocyte esterase	≥10 × 10^7^ CFU/L *Klebsiella* pneumonia ≥10 × 10^7^ CFU/L *Proteus mirabilis*

Abbreviation: CFU, colony-forming units.

### Missing Data

In the clinical model, data were missing for 1 predictor variable (sex and circumcision status) in 4 of 2561 participants (<1%). For the clinical and laboratory model, urine dipstick results were available for 898 of 1723 participants (52%) whose predicted UTI risk exceeded 2% based on clinical predictors (and thus would have been included in modeling). As this was an observational cohort, the decision to obtain urine testing was at the discretion of the clinical team. Follow-up survey data to assess for missed UTIs were obtained for 1099 of 1404 participants (78%) who did not undergo urine testing at the index visit, leading to complete data for 2256 participants (88%). The characteristics of survey responders and nonresponders were comparable (eTable in Supplement 1).

### Model Performance

Model performance was evaluated at predefined risk thresholds. We evaluated performance at 2% for the clinical model and 5% for both models.

#### Clinical Model at 2% Threshold

At the 2% threshold, the clinical model demonstrated high sensitivity (96.4%; 95% CI, 92.8%-99.1%) but low specificity (34.1%; 95% CI, 32.2%-35.9%). The positive predictive value (PPV) was 6.2%, and the negative predictive value (NPV) was 99.5%.

#### Clinical Model at 5% Threshold

At the 5% threshold, the clinical model showed improved specificity (73.8%; 95% CI, 72.1%-75.5%) with reduced sensitivity (82.0%; 95% CI, 74.8%-88.3%). The PPV was 12.5%, and the NPV was 98.9%.

#### Clinical and Dipstick Model at 5% Threshold

The clinical and dipstick model demonstrated high sensitivity (94.0%; 95% CI, 89.0%-98.0%) and specificity (86.9%; 95% CI, 84.5%-89.2%) at the 5% threshold. The PPV was 47.5%, and the NPV was 99.1%.

#### Discrimination and Calibration

**Figure 2.**
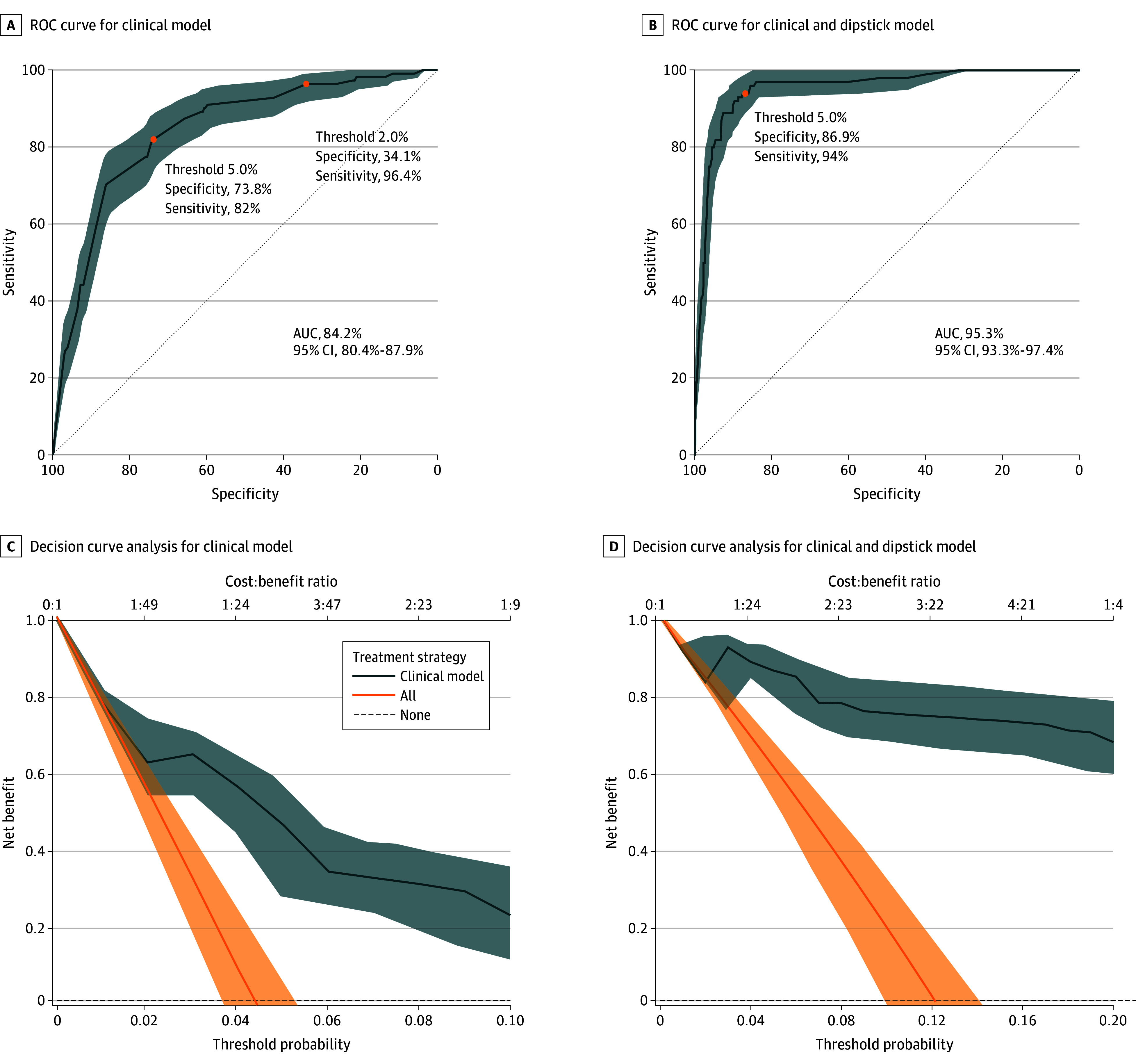
Receiver Operating Characteristic (ROC) Curves and Decision Curve Analyses for Clinical and Clinical and Dipstick UTICalc Models A higher net benefit indicates greater clinical utility. AUC indicates area under the curve.

Discrimination was strong for both models, with AUROC values of 0.84 (95% CI, 0.80-0.88) for the clinical model and 0.95 (95% CI, 0.93-0.97) for the clinical and dipstick model. Brier scores were low for both models, reflecting good overall predictive accuracy. Calibration-in-the-large was close to 0, and both models had calibration slopes less than 1, suggesting some overfitting. The observed-to-expected event ratio indicated underprediction with the clinical model and good calibration with the clinical and dipstick model. Full performance metrics are presented in Table 3, with ROC curves shown in Figure 2.

**Table 3.** Model Performance Metrics

Metric	Model, value (95% CI)		
	Clinical (n = 2557)		Clinical and dipstick, 5% risk threshold (n = 895)
	2% Risk threshold	5% Risk threshold	
Discrimination			
Sensitivity, %	96.4 (92.8-99.1)	82 (74.8-88.3)	94.0 (89.0-98.0)
Specificity, %	34.1 (32.2-35.9)	73.8 (72.1-75.5)	86.9 (84.5-89.1)
Positive predictive value	6.2 (5.9-6.5)	12.5 (11.3-13.7)	47.5 (43.1-52.2)
Negative predictive value	99.5 (99.0-99.9)	98.9 (98.5-99.3)	99.1 (98.4-99.7)
Calibration^a^			
Area under the receiver operating characteristic curve	84.1 (80.4-87.9)		95.3 (93.3-97.4)
Calibration in the large^b^	0.01		0.05
Calibration slope^c^	0.11 (0.09-0.13)		0.06 (0.05-0.07)
Observed-to-expected ratio^d^	0.84 (0.69-0.99)		0.97 (0.79-1.17)
Brier score^e^	0.04 (0.03-0.05)		0.05 (0.04-0.06)

^a^ Calibration values for the clinical model apply to both the 2% and 5% risk thresholds.

^b^ Compares mean predicted risk to mean observed outcomes; a well-calibrated model has calibration-in-the-large close to zero.

^c^ Evaluates how well the spread of predicted risks matches observed outcomes; a slope of 1 indicates perfect calibration.

^d^ Compares the number of observed events with the number of expected events by the model; a ratio of 1 would indicate perfect agreement.

^e^ Measure of overall accuracy of probabilistic predictions, calculated as the mean squared difference between predicted probabilities and actual outcomes; lower scores are better.

### Clinical Utility

#### Decision Curve Analysis

Decision curve analysis demonstrated clinical utility across a range of threshold probabilities. As shown in Figure 2, the clinical model was associated with a higher net benefit than either the treat-all or treat-none strategies across threshold probabilities ranging from 0.01 to 0.08, with the greatest clinical advantage observed between 0.02 and 0.06. Similarly, the clinical and dipstick model was associated with superior net benefit over the same comparators from threshold probabilities of 0.01 to 0.18, with particularly robust benefit in the 0.04 to 0.12 range. These results suggest that both models could aid clinical decision-making by identifying patients more likely to benefit from intervention, with the clinical and dipstick model demonstrating an extended range of utility.

#### Comparison of UTICalc Urine Testing Recommendations vs Clinician Judgement

In our sample, clinicians obtained urine testing for 1157 children, missing 2 cases of UTI. This yielded a sensitivity of 98.2% (95% CI, 93.7%-99.5%) and a specificity of 57.3% (95% CI, 55.4%-59.3%). When compared with UTICalc recommendations at a 2% risk threshold, the model would recommend urine testing in 1719 cases, missing 4 UTIs but increasing the number of urine tests by 565. At a 5% risk threshold, UTICalc would have recommended testing in only 731 cases, missing 20 UTIs. This represents a substantial reduction in testing volume, albeit with a trade-off in sensitivity.

## Discussion

This multicenter diagnostic study prospectively provides the final stage of external validation for UTICalc in young febrile children. We found strong discrimination for the model, which was improved when incorporating dipstick data. This study supports integration into clinical care as a tool for diagnostic stewardship in pediatric care.

Decision curve analysis supported the utility of both UTICalc models across relevant thresholds. However, clinician judgment demonstrated higher sensitivity in the high-volume tertiary centers where this study was conducted, reflecting pediatric emergency medicine practitioner expertise. Because most children presenting for acute care are evaluated outside tertiary centers,^27^ possibly with limited pediatric expertise and uncertain follow-up, using a 5% risk threshold—despite reducing testing—may not be ideal due to reduced sensitivity. UTICalc may serve as a useful adjunct for clinicians with less pediatric experience or in cases of diagnostic uncertainty, with lower risk thresholds (eg, 2%) potentially more appropriate for patients with persistent symptoms or anticipated barriers to follow-up.

The observed UTI prevalence of 4% in our cohort is consistent with previously reported risk estimates in this population, which ranges from 3% to 11%.^2,28,29^ Model performance in our study was comparable with the original derivation and validation study published in 2018, which reported an AUROC of 0.80 in the clinical model and 0.97 in the clinical and dipstick model. Low PPV in our sample is reflective of low disease prevalence.

Since the internal validation of UTICalc,^14^ 2 external validation studies^16,30^ have been conducted with important limitations. In 2021, Boon et al^30^ evaluated UTICalc in a small subgroup (n = 96) with only 4 positive UTI cases and resultant large confidence intervals in their conclusions, limiting interpretation. More recently, Smith et al^16^ conducted a retrospective case-control study comparing UTICalc version 1.0 with the current version 3.0 (excluding race as a predictor). While they demonstrated reasonable discrimination (AUROC, 73.8; 95% CI, 68.7-78.8), this study was limited by the retrospective design and inclusion of only those children who had both a urinalysis and urine culture performed, potentially introducing significant selection and verification bias. Given that UTICalc is designed to identify which undifferentiated, febrile child warrants urine testing (and potentially empiric treatment), prospective validation in this population is essential. Our study provides clinical evidence of its performance in the population for which it was designed.

Strengths of this study include its prospective design, large sample size, and follow-up to identify missed cases. Inclusion of 2 distinct settings, one where UTICalc is rarely used, enhances the generalizability of findings. The model’s performance was rigorously evaluated using TRIPOD-recommended metrics.^23^

### Limitations

This study had several limitations. Maximum recorded temperature, duration of fever, and prior history of UTI were obtained from caregivers; however, any minor discrepancies between caregiver report and clinician documentation are unlikely to meaningfully affect results, as these elements are routinely obtained during clinical history taking. Missing urinalysis data, while reflective of the realities of clinical decision-making, may have introduced verification bias. We mitigated this by implementing a follow-up strategy to identify missed UTI diagnoses; however, follow-up data were not available for a small proportion of participants. UTIs may have been missed if patients were treated for other apparent infections (eg, acute otitis media). We were also unable to validate the Gram stain model, as neither participating site reported Gram stain results. Validation of the hemocytometer method was limited due to inconsistencies in white blood cell unit conversions. Furthermore, interrater reliability and clinical acceptability of the tool were not assessed in this study, which may affect its practical utility in clinical settings.

## Conclusions

In this multicenter prospective cohort study of 2561 febrile children aged 2 to 24 months presenting to the ED, UTICalc demonstrated strong discrimination, calibration, and clinical utility. Although UTICalc did not outperform clinician performance at the high-volume tertiary pediatric centers in which the study was conducted, the prospective design of the study allows for generalizability of its discrimination, overall accuracy, and net benefit. These findings support the use of UTICalc to guide UTI evaluation and management in young children. Future research should assess UTICalc performance across varying levels of clinician pretest suspicion and evaluate earlier integration into ED workflows, potentially using only historical and clinical features, to support earlier risk stratification and streamline urine collection.

## Notes

Supplement 1. **eTable.** Characteristics of Survey Responders and Nonresponders

Supplement 2.
Nonauthor Collaborators

Supplement 3.
Data Sharing Statement
