# Supplementary material for: Diagnosing Urinary Tract Infection in Young Febrile Children in the Emergency Department
Source: JAMA Netw Open. 2026 Mar 13;9(3):e261741. doi: 10.1001/jamanetworkopen.2026.1741 (PMC12988452; doi:10.1001/jamanetworkopen.2026.1741)
Supplement: Supplement 1. — eTable. Characteristics of Survey Responders and Nonresponders [file jamanetwopen-e261741-s001.pdf]

Supplemental Online Content

Kinlin C, Gravel J, Barrowman N, et al; Pediatric Emergency Research Canada (PERC) Network. Diagnosing urinary tract infection in young febrile children in the emergency department. *JAMA Netw Open.* 2026;9(3):e261741. doi:10.1001/jamanetworkopen.2026.1741

eTable. Characteristics of Survey Responders and Nonresponders

This supplemental material has been provided by the authors to give readers additional information about their work.

© 2026 Kinlin C et al. *JAMA Network Open*.

eTable. Characteristics of Survey Responders and Nonresponders

**Characteristic**	**N**	**Overall** N = 1,404[1]	**Non-responders** N = 305[1]	**Responders** N = 1,099[1]
**Recruitment Site**	1,404			
cheo		777 (55%)	152 (50%)	625 (57%)
ste_justine		627 (45%)	153 (50%)	474 (43%)
**Age group (months)**	1,404			
2 to 11.99		555 (40%)	123 (40%)	432 (39%)
12 to 23.99		849 (60%)	182 (60%)	667 (61%)
**Age (months, Median [IQR])**	1,404	13.4 [9.3, 18.3]	13.7 [9.4, 17.9]	13.4 [9.3, 18.4]
**Sex and circumcision status**	1,403			
Male, circumcised		307 (22%)	70 (23%)	237 (22%)
Male, uncircumcised		501 (36%)	98 (32%)	403 (37%)
Female		595 (42%)	137 (45%)	458 (42%)
NA		1	0	1
**Personal history of UTI**	1,404			
Yes / Oui		53 (3.8%)	11 (3.6%)	42 (3.8%)
No / Non		1,351 (96%)	294 (96%)	1,057 (96%)
**Family history of UTI**	1,404			
Yes / Oui		477 (34%)	102 (33%)	375 (34%)
No / Non		889 (63%)	192 (63%)	697 (63%)
Unknown / Je ne sais pas		38 (2.7%)	11 (3.6%)	27 (2.5%)
**Maximum temperature >=39 degrees**	1,404			
<39		432 (31%)	88 (29%)	344 (31%)
>=39		972 (69%)	217 (71%)	755 (69%)
**Duration of fever >=48 hours**	1,404			
<48h		573 (41%)	110 (36%)	463 (42%)
>=48h		831 (59%)	195 (64%)	636 (58%)
**Fever source Identified**	1,404	1,340 (95%)	298 (98%)	1,042 (95%)

[1]n (%); Median [Q1, Q3]

© 2026 Kinlin C et al. *JAMA Network Open*.
