# Supplementary material for: Diagnosing Urinary Tract Infection in Young Febrile Children in the Emergency Department
Source: JAMA Netw Open. 2026 Mar 13;9(3):e261741. doi: 10.1001/jamanetworkopen.2026.1741 (PMC12988452; doi:10.1001/jamanetworkopen.2026.1741)
Supplement: Supplement 2. — Nonauthor Collaborators [file jamanetwopen-e261741-s002.pdf]

Supplemental Online Content: Nonauthor Collaborators
*First name, last name, and suffix (if applicable) are required and will appear in PubMed.

*Group Name(s): Pediatric Emergency Research Canada (PERC) Network							
***First Name and Middle Initial(s)**	***Last Name**	***Suffix (eg, Jr, III)**	Academic Degrees	Institution	Location (city, state/province, country)	Role or Contribution, eg, chair, principal investigator	Group (if more than 1 Group listed in the byline) and/or Subgroup (eg, Steering Committee)
Samina	Ali		MD	University of Alberta	Edmonton, Alberta, Canada	Chair	
Naveen	Poonai		MD	Western University	London, Ontario, Canada	Vice Chair	
Vikram	Sabhaney		MD	University of British Columbia	Vancouver, British Columbi	Executive Member	
Bruce	Wright		MD	University of Alberta	Edmonton, Alberta, Canada	Executive Member	
Gabrielle Christine	Freire		MD	University of Toronto	Toronto, Ontario, Canada	Executive Member	
Henry	Li		MD	University of Alberta	Edmonton, Alberta, Canada	Executive Member	
Tyrus	Crawford		BSoc.Sci.	University of Ottawa	Ottawa, Ontario, Canada	Executive Member	
Andrea	Eaton		BScN, BPE.	University of Alberta	Edmonton, Alberta, Canada	Executive Member	
