# Supplementary material for: Diagnosing Urinary Tract Infection in Young Febrile Children in the Emergency Department
Source: JAMA Netw Open. 2026 Mar 13;9(3):e261741. doi: 10.1001/jamanetworkopen.2026.1741 (PMC12988452; doi:10.1001/jamanetworkopen.2026.1741)
Supplement: Supplement 3. — Data Sharing Statement [file jamanetwopen-e261741-s003.pdf]

Data Sharing Statement

Kinlin. Diagnosing Urinary Tract Infection in Young Febrile Children in the Emergency Department. *JAMA Netw Open*. Published March 13, 2026.
doi:10.1001/jamanetworkopen.2026.1741

Data
Data available: No
